# Profiling investor behavior in the Malaysian derivatives market using K-means clustering

**DOI:** 10.3389/frai.2025.1640776

**Published:** 2025-09-17

**Authors:** Eng Hao Louis Tan, Yaman Hamed, Hanita Daud, Mohd Amirul Faiz Abdul Wahab, Ahmad Amirul Adlan Azhar, Sieow Yeek Tan

**Affiliations:** ^1^Department of Applied Sciences, Intelligent Asset Reliability Centre, Institute of Emerging Digital Technologies, Universiti Teknologi PETRONAS, Seri Iskandar, Malaysia; ^2^Bursa Malaysia Berhad, Kuala Lumpur, Malaysia

**Keywords:** clustering, K-means, decision trees, trading behavior, derivatives, investors behavior

## Abstract

This study investigates the trading behaviors of Malaysian derivatives traders using a comprehensive dataset from Bursa Malaysia with K-means clustering, representing one of the first AI applications to derivatives market segmentation. The analysis encompassed over 11 million trade records for FCPO and FKLI derivatives from January to December 2022. Six key features were engineered to segment derivative traders: Total Number of Trades, Total Traded Amount, Overall Realized Profit, Average ROI, Maximum Account Vintage (trader experience in years), and Median Holding Days (typical position duration). Inverse Hyperbolic Sine transformation was applied to address extreme outliers, ensuring robust feature scaling. K-means clustering identified five distinct profiles: “High-Frequency, High-Risk Derivative Traders with Consistent Losses,” “Conservative, Steady-Growth Derivative Trader,” “High-Frequency, High-Yield Derivative Traders,” “Conservative, Low-Yield Derivative Traders,” and “Cautious, Low-Activity Novice Derivative Traders.” Decision tree classifiers validated these clusters through interpretable splitting conditions. These profiles enable targeted risk management strategies, personalized trading services, and evidence-based regulatory policies for derivatives markets and future research.

## Introduction

Derivatives are financial instruments whose value derives from underlying assets like commodities, stocks, or indices, used for hedging risks, speculation, and portfolio enhancement. Malaysia’s most actively traded derivatives include Futures Crude Palm Oil (FCPO), linked to crude palm oil prices, and Futures Kuala Lumpur Index (FKLI). FCPO is a commodity-based derivative linked to the price of crude palm oil, a significant export commodity for Malaysia, making it particularly attractive for participants in the agricultural and commodities sectors (Rizal et al., 2023; Jamak, 2018). FKLI, is an index-based derivative that tracks the performance of the Bursa Malaysia Kuala Lumpur Composite Index, which represents the Malaysian stock market’s benchmark index (Jamak, 2018; Seng and Thaker, 2018). These derivatives attract diverse participants who employ varied investment strategies, with 25% of publicly listed firms on Bursa Malaysia using derivatives for hedging from 2003 to 2007 (Seng and Thaker, 2018).

In financial markets, customer segmentation divides populations into distinct groups based on common characteristics, enabling tailored services that help provide improved investment strategies (Clark-Murphy and Soutar, 2005; Kashwan and Velu, 2013; Wood and Zaichkowsky, 2004). Trading segmentation identifies trader typologies based on strategies, risk tolerance, and behavioral patterns which include high-frequency traders, long-term investors, and hedgers (Keller and Siegrist, 2006; Berglöf, 1985). While extensive research exists on investor segmentation in stock markets, limited studies have explored segmentation in derivatives markets. Given the differences in underlying assets, market behaviors, and risk dynamics, studying these segments is critical for understanding trading patterns in the derivatives market (Subeesh and Liya, 2024; Somanathan and Nageswaran, 2015). The resulting trader profiles can help regulators determine when trader positions become large enough to potentially manipulate prices away from legitimate supply and demand conditions (Sanders et al., 2004).

This study applies K-means clustering to historical FCPO and FKLI trade data to identify distinct derivatives trader clusters, representing one of the first comprehensive applications of machine learning techniques specifically to derivatives trader segmentation. MacQueen in 1967 introduced the K-means algorithm as a method for partitioning observations into k clusters, establishing the mathematical framework that remains fundamental to modern clustering applications (MacQueen, 1967). K-means efficiently partitions data into groups by minimizing intra-cluster variance, providing interpretable results for financial market analysis (Wood and Zaichkowsky, 2004; Fawaid Ridwan and Supian, 2021; Kalra Sahi and Arora, 2012). The categorical variables within clusters were analyzed, and performance differences between clusters were investigated to provide insights into the behavior of derivatives market traders. The analysis also considered potential implications for trading strategies and risk management practices that could benefit market stakeholders. A novel decision tree validation approach is developed to uniquely characterize cluster membership, providing actionable behavioral insights for market stakeholders. This approach provides better identification compared to the current usage of ANOVA and Hypothesis testing that is used to highlight the differences between the resulted clusters, which does not require any normality assumptions and/or linearity.

The remainder of this paper is organized as follows: Section 2 presents a literature review of derivatives trading and clustering methodologies. Section 3 details data collection, feature engineering, and transformation techniques. Section 4 includes a detailed analysis of the K-means clustering outcomes across continuous and categorical variables. Section 5 explores cluster characteristics and suggests the main criteria of the trader clusters using decision tree node splits, validated through boxplot distributions. Section 6 summarizes the findings and offers future direction.

## Literature review

Research on derivatives investor behavior has revealed distinct trading preferences and patterns. Yuen (2013) suggested that investment experience directly correlates with average returns and trading performance, while also identifying heterogeneous investor profiles characterized by different risk tolerances, holding periods, and product preferences. The shift toward online derivatives trading has also influenced investor behavior, with studies showing increased trading frequency and altered decision-making patterns among participants using digital platforms (Yuen, 2013). Shi et al. (2018) claimed that certain traders participate in specific transaction patterns, and only some trading characteristics of certain traders in a time window will reflect the trading behavior patterns. This suggested distinct behavioral clusters within derivatives markets.

Related studies that used trade data for clustering investors into significant groups were reviewed to demonstrate the application of clustering methodologies in financial market traders. Notably, there remains a significant shortage of research applying clustering techniques to derivatives market data. This study addresses this research gap by utilizing trade data extracted specifically from derivatives markets to identify distinct investor profiles, thereby extending the application of clustering methodologies beyond the commonly studied equity markets. Additionally, an innovative decision tree validation methodology for post-clustering validation and characterization provides decision rules for each identified cluster, representing a novel contribution to financial market segmentation research. The related work to this research is summarized in Table 1.

**Table 1:** Summary of related work.

Authors	Year	Dataset	Algorithm	Clusters	Ref.
Shin & Sohn	2004	2,999 customers (HTS & assisted trading)	K-means, SOM, Fuzzy K-means	Normal (95%), Best (3%), VIP (0.2–0.5%)	Shin and Sohn (2004)
Wang et al.	2009	30,287 investors	Voting K-means	Conservative (52%), Speculative (27%), Moderate (21%)	Wang et al. (2009)
Goshima et al.	2019	144 trading desks	Hierarchical Clustering	HFT Market Makers (8%), Opportunistic HFT (17%), Middle-Frequency (74%) + Low-Frequency (manual)	Goshima et al. (2019)
Thompson et al.	2021	52,025 accounts	K-prototype	Active (19%), Early Savers (36%), Just-In-Time (27%), Older (7%), Systematic Savers (12%)	Thompson et al. (2021)
Hwang et al.	2024	339,007 investors, 955,035 entries	Gaussian Mixture Model	8 clusters (unspecified)	Hwang et al. (2024)
Vlahavas et al.	2024	105,589,345 transactions	K-means	Cluster 1 (61.4%), Cluster 2 (19.3%), Cluster 3 (11.5%), Cluster 4 & 5 (~3% each)	Vlahavas et al. (2024)

Shin and Sohn focused on total trade amounts over three months, analyzing representative-assisted trading and the online Home Trading System (HTS) of 2,999 customers (Shin and Sohn, 2004). The authors applied K-means, Self Organizing Maps, and fuzzy K-means as the clustering algorithms. The representative-assisted trading data were described by “total trade amount” and “representative-assisted trade amount.” The online HTS was represented by “total trade amount” and “trade amount in HTS” as the main raw features. The authors identified three clusters, normal customers (95%) (trading below specified thresholds in both trading modes), best customers (3%) (trading at intermediate levels), and VIP Customers (0.2–0.5%) who exhibited the highest trade volumes across both trading modes. The authors introduced a new brokerage commission policy based on the identified clusters for a potential of higher profit.

Wang et al. used the records of 30,287 investors to categorize them into three predefined clusters. The customer purchasing and selling frequency, proportion of transaction amount to total assets, and proportion of deposit to total assets were used as the clustering features (Wang et al., 2009). The authors used voting K-means to categorize the investors into three groups, Conservative Investors (52%), Speculative Investors (27%), and Moderate Investors (21%). Conservative Investors preferred low-risk instruments like time deposits, demonstrating minimal engagement with high-risk products. Speculative Investors favored high-risk financial products across all categories. While Moderate Investors adopted a balanced approach, blending conservative and speculative strategies. The clusters acquired an accuracy of 87% when evaluated using a randomly selected 200 customers from the dataset.

Goshima et al. analyzed 144 trading desks using hierarchical clustering based on four key metrics, Cancellation to Order Ratio, Inventory Ratio, Number of Actions per Stock, and Number of Stocks per Trading Desk (Goshima et al., 2019). Their analysis initially yielded ten clusters, which they subsequently consolidated into three main trader categories with distinctive characteristics. The High-Frequency Trader Market Makers (8%) exhibited the highest cancellation-to-order ratios combined with minimal inventory holdings. Investors in this group are typical for high-frequency limit order strategies. The Opportunistic High-Frequency Traders (17%) displayed either elevated cancellation-to-order ratios or reduced inventory ratios, but not both simultaneously. The Middle-Frequency Traders (74%) maintained moderate values across both the Cancellation-to-Order Ratio and the Inventory Ratio. Additionally, they included a fourth category outside their clustering analysis (Low-Frequency Traders) which comprised of additional 2,177 trading desks with distinctly different trading patterns.

Thompson et al. used K-prototype clustering to segment 52,025 accounts based on investor demographics, trading frequency, and traded amount. Their analysis resulted in five clusters: Active Traders (19%), engaging in frequent, high-volume manual trades with moderate risk tolerance; Early Savers (36%), younger individuals relying on systematic transactions with minimal trading activity; Just-In-Time (27%), characterized by infrequent, small manual trades with slightly lower risk tolerance; Older Investors (7%), who prioritized withdrawals and dividends and exhibited the lowest risk tolerance; and Systematic Savers (12%), who employed periodic, systematic trading with a similar risk profile to active traders (Thompson et al., 2021).

Hwang et al. conducted an investor clustering analysis using a substantial dataset comprising 339,007 investors with 955,035 data entries spanning January 2016 to December 2020. They utilized 23 variables across five categories: account overview, buy/sell orders, deposits/withdrawals, transaction proportions, and transaction details. The researchers identified eight clusters by employing the Gaussian Mixture Model. The resulting clusters exhibited varying characteristics, including differences in average balances, trading volumes, transaction values, turnover rates, and deposit/withdrawal patterns (Hwang et al., 2024). Notably, some clusters demonstrated inverse relationships between account balance and trading activity, while others showed distinctive patterns in terms of transaction frequency and value.

Vlahavas et al. analyzed Bitcoin transaction behavior using K-means clustering on a comprehensive dataset comprising 105,589,345 transactions to identify distinct user behavioral patterns in cryptocurrency markets (Vlahavas et al., 2024). Their analysis resulted in five clusters: Cluster 1 (61.4%), Cluster 2 (19.3%), Cluster 3 (11.5%), and Clusters 4 and 5 (approximately 3% each). While the study did not provide detailed names for each cluster, it demonstrated the effectiveness of unsupervised clustering techniques in revealing hidden patterns within blockchain transaction data, providing insights into the heterogeneous nature of cryptocurrency market participants (Vlahavas et al., 2024).

## Methodology

**Figure 1:**
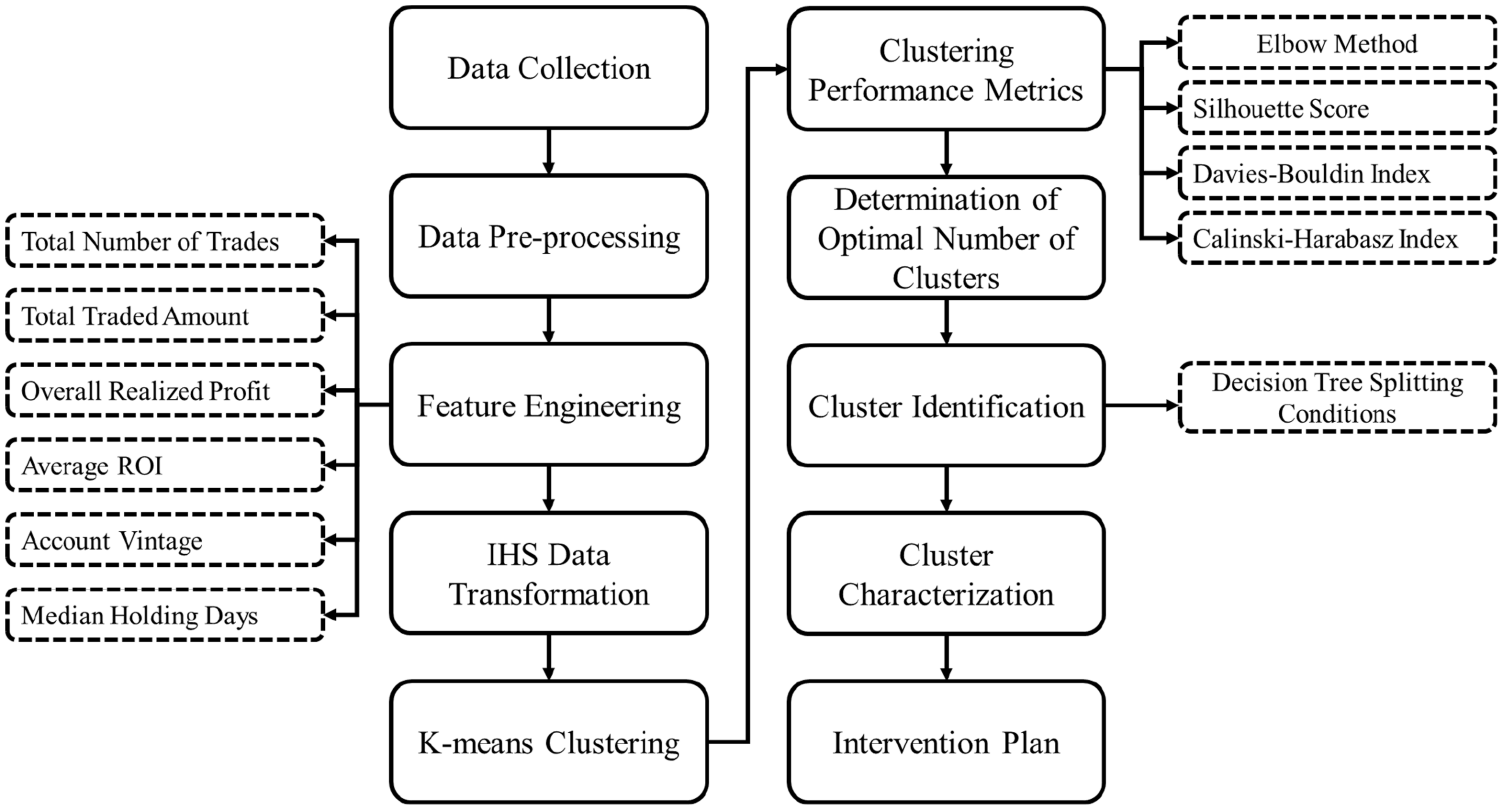
Methodology flow chart.

The stepwise framework of the proposed methodology is illustrated in Figure 1.

### Data description

The data used for this analysis were provided by BURSA Malaysia. The dataset comprises 11,222,606 rows of trade data collected between January 2022 and December 2022, covering two product codes, FCPO and FKLI. The records are stored in a structured SQL database. The data was filtered to include only traders registered in Malaysia, which reduced it to 11,117,203 rows, removing approximately 1% of the original data. To facilitate efficient data management and querying, a unique primary key was created by hashing a combination of three attributes: investor ID, broker participant ID, and account ID. The hierarchical structure prioritizes the broker participant ID, followed by the investor ID, and finally the account ID. This process generated 9,852 unique primary hash keys, representing 9,852 unique accounts, 8,816 unique traders, and 13 broker participants.

**Figure 2:**
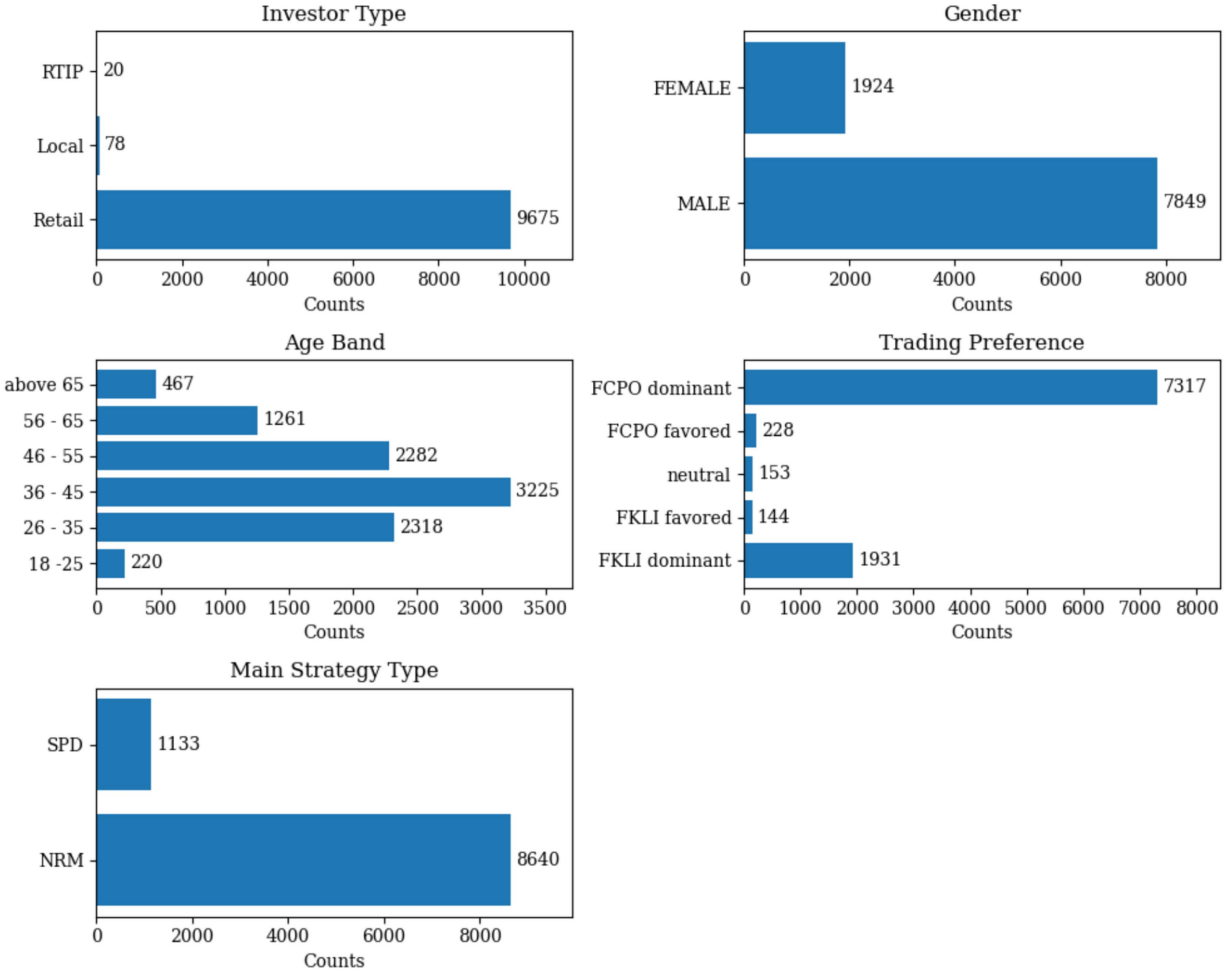
Distribution of categorical variables across the dataset of 9,773 derivatives traders (79 traders were removed due to no realized profit).

The dataset was further categorized based on the frequency of trade records for FCPO and FKLI to capture the trading preferences. Each unique primary hash key was classified into one of five categories: FCPO dominant, FCPO favored, neutral, FKLI favored, and FKLI dominant. Each transaction is made using one of two different trading strategy types, SPD (Derivatives that are based on the spread between the prices of two or more assets) and NRM (derivatives with one directional to buy/sell contracts). Therefore, the most frequent strategy type used by each unique primary hash key was recorded and associated with the respective account. Five categorical variables describe investor traits in the dataset, age group, gender, investor type, trade product preference, and most used strategy type. The characteristics of the studied data are illustrated in Figure 2.

### Feature engineering and transformation

Six features were generated to analyze the trading behavior of traders. Each feature was designed to capture a distinct aspect of the trading activities. The generated features are the Total Number of Trades, Total Traded Amount, Overall Realized Profit, Average Return on Investment (ROI), Maximum Account Vintage, and Median Holding Days. Each feature is derived from the dataset and grouped by the unique primary hash key index, ensuring that they accurately represent individual trading behavior.

The Total Number of Trades corresponds to the total count of trade records in the dataset associated with each unique primary hash key. The Total Traded Amount is calculated as the cumulative sum of the trade values for all buy and sell transactions grouped by the unique primary hash key. The Overall Realized Profit represents the net profit or loss achieved by each trader. It is calculated by subtracting the total bought amount from the total sold amount for matched trades, where the quantities of bought and sold transactions align. A positive value indicates a net profit, while a negative value signifies a loss. Similarly, Average ROI is calculated as the realized profit divided by the total bought amount for matched trades. This metric provides a normalized measure of profitability, allowing for direct comparisons across traders regardless of the scale of their trading activities. It is particularly useful for identifying efficient traders who achieve high returns with limited resources. The Maximum Account Vintage reflects the longevity of a trader’s account and is calculated as the difference between the last recorded trade date and the account creation date (expressed in years). The account vintage provides insights into the trader’s experience and commitment over time to distinguish between newer participants and long-standing traders who may exhibit more stable or sophisticated trading behaviors. Finally, Median Holding Days capture the typical duration for which a trader holds a trade position before closing it. This is calculated as the median of the holding durations for all matched trades associated with each unique primary hash key, where the holding duration is the time elapsed between the creation of the buy and sell transactions. It offers a view into the trader’s trading strategy, revealing whether they tend toward short-term trading for quick gains or long-term investments that aim for sustained returns. The formulated engineered features are summarised in Table 2.

**Table 2:** Formulated engineered features.

Feature name	Description	Formula
Total number of trades	Total count of trades per trader (per hash key)	$N_{i}=\sum_{j=1}^{n_{i}}1\;$
Total traded amount	The sum of all trade values (buy and sell) for each trader	$T_{i}=\sum_{j=1}^{n_{i}}P_{\mathrm{ij}}\times Q_{\mathrm{ij}}$
Overall realized profit	Net profit/loss from matched trades	${\mathrm{ORP}}_{i}=\sum_{k=1}^{m_{i}}S_{\mathrm{ik}}-B_{\mathrm{ik}}$
Average ROI	Normalized profitability measure per trader	${\mathrm{ROI}}_{i}=\frac{{\mathrm{ORP}}_{i}}{\sum_{k=1}^{m_{i}}B_{\mathrm{ik}}}$
Maximum account vintage	Trader’s account age in years	$V_{i}=\frac{{\mathrm{LTD}}_{i}-{\mathrm{ACD}}_{i}}{365}$
Median holding days	Median number of days trades are held before selling.	$H_{i}=\text{Median}\;(SD_{\mathrm{ik}}-BD_{\mathrm{ik}})$

i : index of trader, j : index of trade, k : index of matched trade, $n_{i}$ : number of rows for trader i , $m_{i}$ : total number of match traders for trader i , $P_{\mathrm{ij}}$ : the price of trade j for trader i , $Q_{\mathrm{ij}}$ : quantity of trade j , $S_{\mathrm{ik}}$ : total value of sell trade, $B_{\mathrm{ik}}$ : total value of buy trade, ${\mathrm{LTD}}_{i}$ : last trade date for trader i , ${\mathrm{ACD}}_{i}$ : account creation date for trader i . ${\mathrm{SD}}_{\mathrm{ik}}$ : sell date of matched trade k , ${\mathrm{BD}}_{\mathrm{ik}}$ : buy date of matched trade k .

A total of 79 unique primary hash keys had no realized profit being computed as no match trades were found. As a result, those hash keys were removed from the analysis which reduced the data from 9,852 to 9,773 unique rows (representing only 0.8% of the dataset). The deleted entries contribute to only 334 total trades in the data (approximately 0.003% of the entire dataset), where 32 out of these 79 unique primary hash keys have exactly one trade from the entire Jan 2022 to Dec 2022 period.

**Figure 3:**
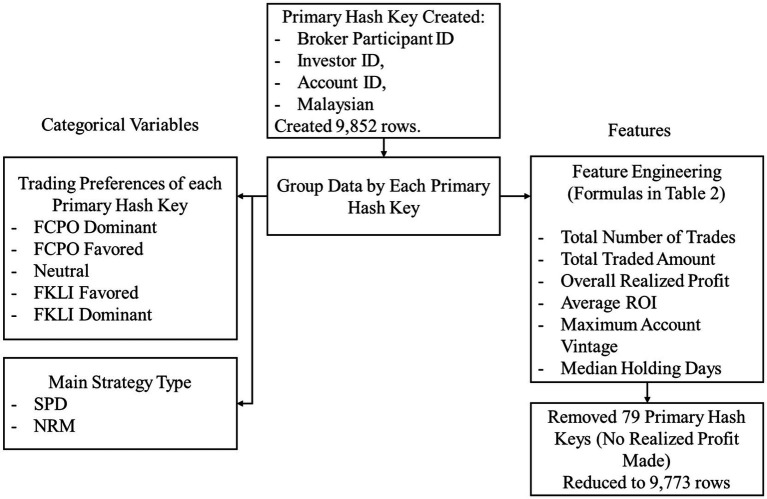
Data preprocessing flow chart of variables and features.

Figure 3 illustrates the preprocessing flow chart of the variables and engineered features.

These features exhibit substantial variability due to the presence of outliers, which can distort the clustering performance of the K-means model. To address this issue, the Inverse Hyperbolic Sine (IHS) transformation was applied to scale down the values of features with extremely large ranges while preserving the overall distribution structure (Equation 1). First, IHS transformation accommodates the full range of financial data including zero and negative values without requiring data truncation or sign loss, unlike log transformation which necessitates positive-only inputs. Second, IHS provides asymptotic behavior that compresses extreme outliers through the asymptotic linearity property, effectively reducing outlier leverage while retaining their directional information (Equation 2). Finally, unlike other normalization techniques such as log transformation or min-max scaling, Hence, IHS maintains the distributional properties and relative ordering of observations while mitigating the disproportionate influence of outliers (Bellemare and Wichman, 2020; Burbidge et al., 1988).

(1) $$\sin h^{-1}x=\ln(x+\sqrt{x^{2}+1})\;$$

(2) $$\lim_{x\to +\infty }\frac{\sin h^{-1}(x)}{\ln(x)}=1\;$$

### K-means clustering

The six generated features were used in the K-means clustering algorithm. K-means is a simple yet powerful clustering algorithm that can be used over a d -dimensional vector $X=[x_{1},x_{2},\ldots ,x_{n}]$ in $R^{d}$ . For a set of n input data points the k-means algorithm begins with the initialization of k centroids, $c_{1},c_{2},\ldots ,c_{k}$ , which are randomly selected from the set of data X . These centroids represent the initial cluster centers (step 1).

Each data point $x_{i}$ in X is then assigned to the nearest centroid $c_{j}$ based on a distance metric (typically the Euclidean distance given in Equation 3) as the assignment step (step 2).

(3) $$d(x_{i},c_{j})={|x_{i}-c_{j}|}_{2}=\sqrt{\sum_{l=1}^{d}{\left(x_{\mathrm{il}}-c_{\mathrm{jl}}\right)}^{2}}$$

Each data point $x_{i}$ is assigned to the cluster $C_{j}$ whose centroid $c_{j}$ is the closest. Once all data points have been assigned to clusters, the centroids $c_{j}$ are recalculated as the mean of the data points in each cluster as given in Equation 4:

(4) $$c_{j}=\frac{1}{|C_{j}|}\sum_{x_{i}\in C_{j}}x_{i}\;$$

Where $|C_{j}|$ is the number of data points in cluster j , and the sum is over all data points assigned to cluster $C_{j}$ . Hence, all the centroids are now updated (step 3). The steps of assignment (step 2) and update (step 3) are repeated until the centroids are no longer changing significantly, or until a maximum number of iterations is reached. The convergence criterion can be the change in the centroids’ positions or the change in the cluster assignments. The objective of k-means is to minimize the within-cluster sum of squares (WCSS), also known as the inertia, as shown in Equation 5:

(5) $$J=\sum_{j=1}^{k}\sum_{x_{i}\in C_{j}}{|x_{i}-c_{j}|}_{2}^{2}$$

The objective function J quantifies the total variance within the clusters, and the k-means algorithm seeks to minimize this value. The final output is a set of k clusters $\left(C_{1},C_{2},\ldots ,C_{k}\right)$ and their corresponding centroids $\left(c_{1},c_{2},\ldots ,c_{k}\right)$ (MacQueen, 1967).

## Results and discussion

**Figure 4:**
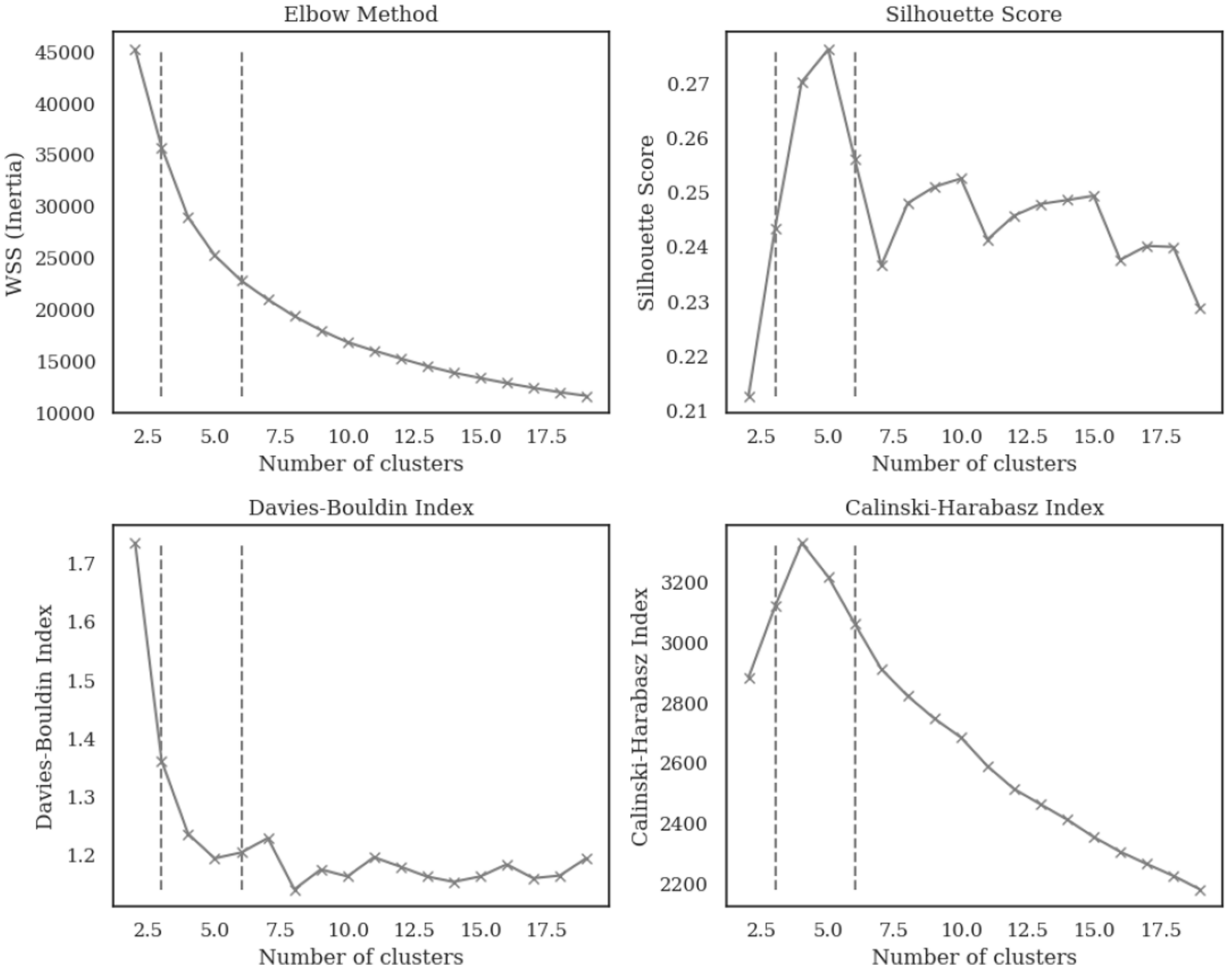
Cluster validation metrics for determining optimal number of clusters.

**Figure 5:**
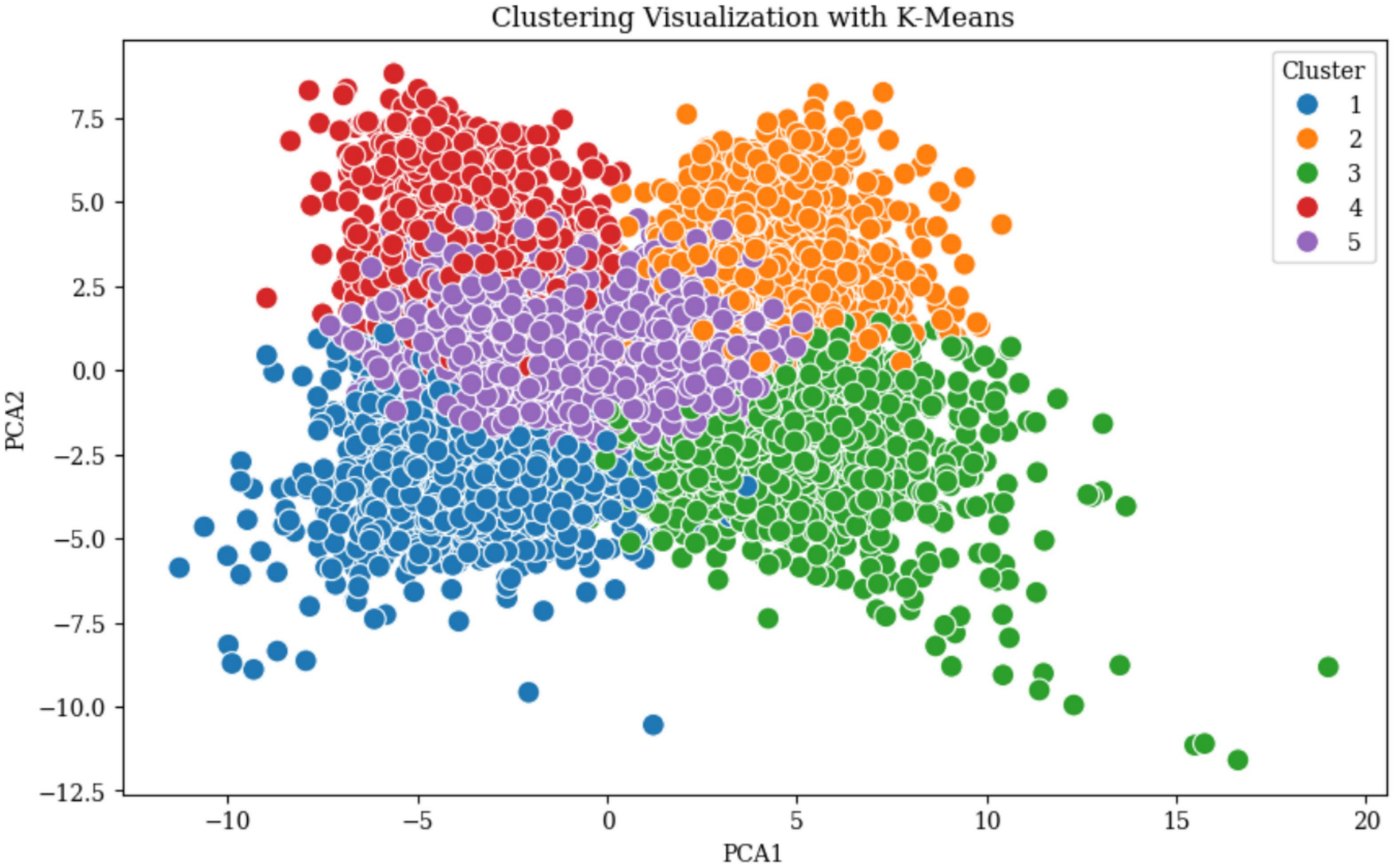
K-means clustering results visualized in two-dimensional PCA space.

**Figure 6:**
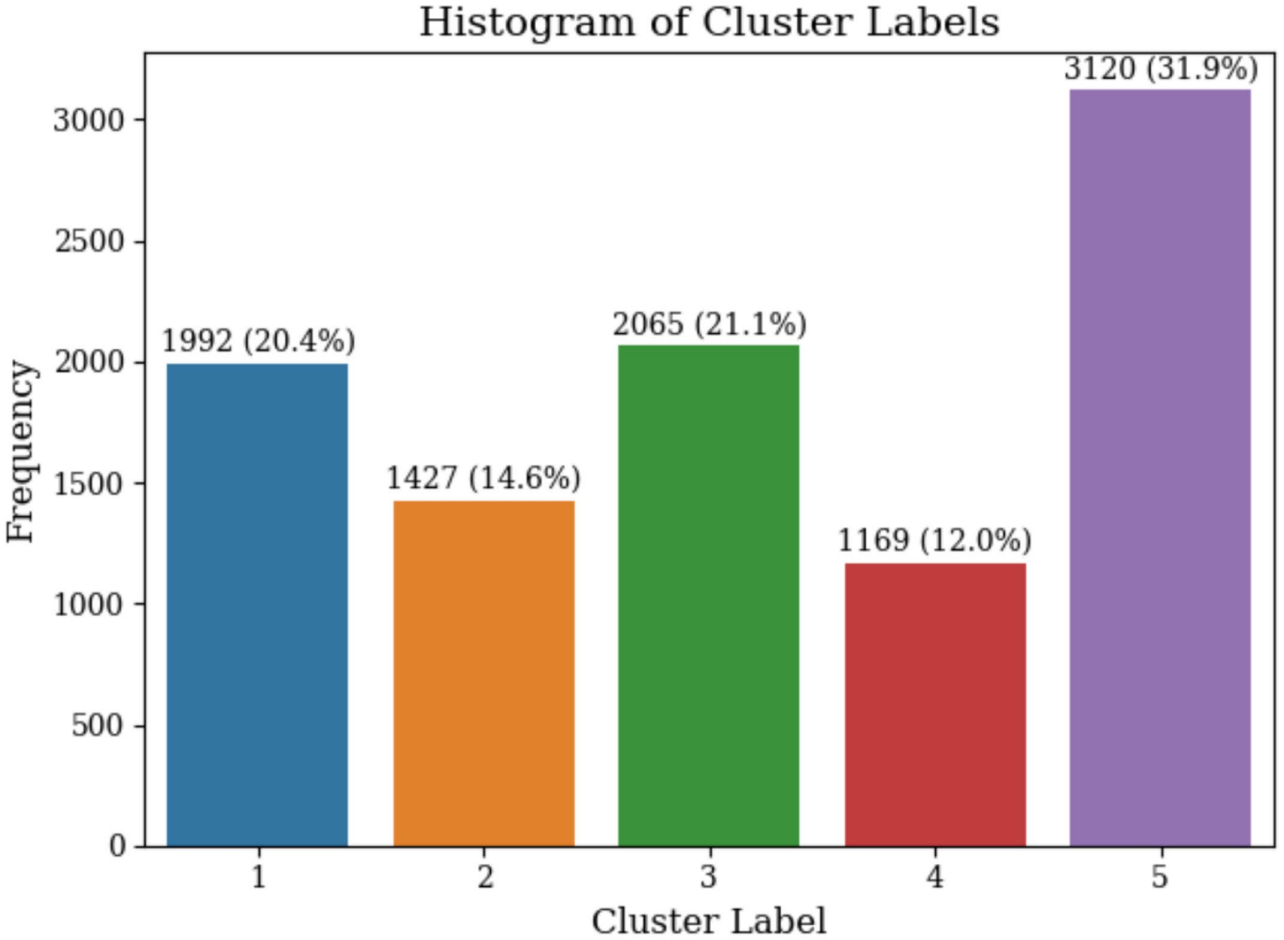
Cluster size distribution showing trader count and percentage for each identified cluster.

The optimal number of clusters (k) was determined using four methods, the Elbow Method, Silhouette Score, Davies-Bouldin Index, and Calinski-Harabasz Index (Rousseeuw, 1987; Davies and Bouldin, 2009; Caliński and Harabasz, 1974). These methods collectively identified the optimal range of k from 3 to 6, where the Elbow Method shows a noticeable bend at k = 5, the Silhouette Score reaches a local maximum, the Davies-Bouldin Index achieves a local minimum value, and the Calinski-Harabasz Index demonstrates high values at this point as illustrated in Figure 4. The convergence of these four indicators at k = 5 provides strong statistical evidence for this optimal cluster number. The five identified clusters are well-separated in the reduced two-dimensional PCA space as illustrated in Figure 5. The distribution of data points among the five identified clusters is given in Figure 6.

**Figure 7:**
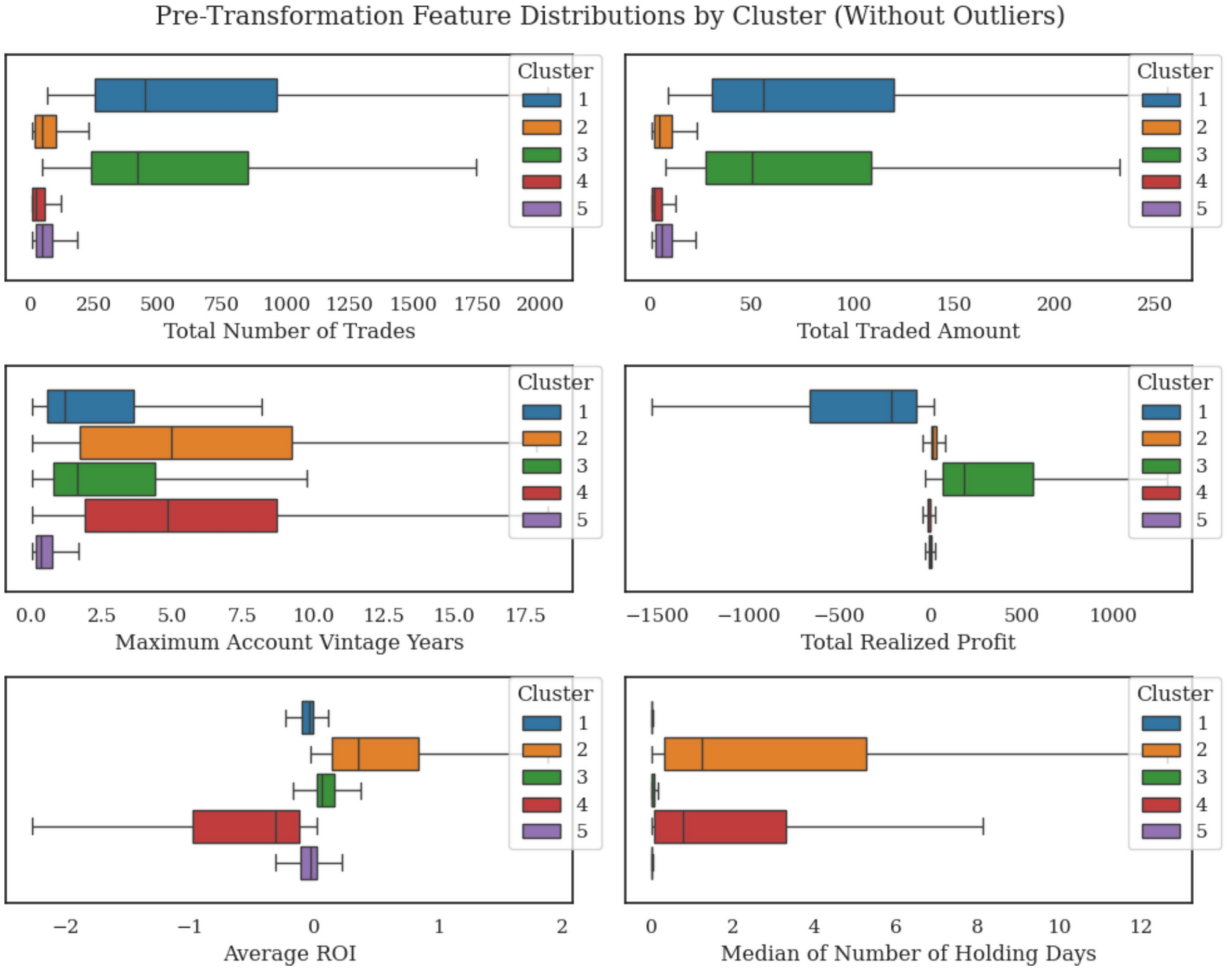
Distribution of six engineered features across the five trader clusters (outliers removed for clarity).

Figure 7 shows the distributions of the six features across the five clusters. The Total Number of Trades and Total Traded Amount exhibit wide ranges across clusters, with clusters 1 and 3 showing higher medians compared to clusters 2 and 4. Similarly, Maximum Account Vintage Years in clusters 2 and 4 demonstrate wider interquartile ranges compared to others. The Total Realized Profit and Average ROI in clusters 1 and 4 display more negative values, while cluster 3 exhibits higher positive medians. The Median Holding Days feature remains relatively low across all clusters, with slightly longer durations observed in clusters 2 and 4.

**Figure 8:**
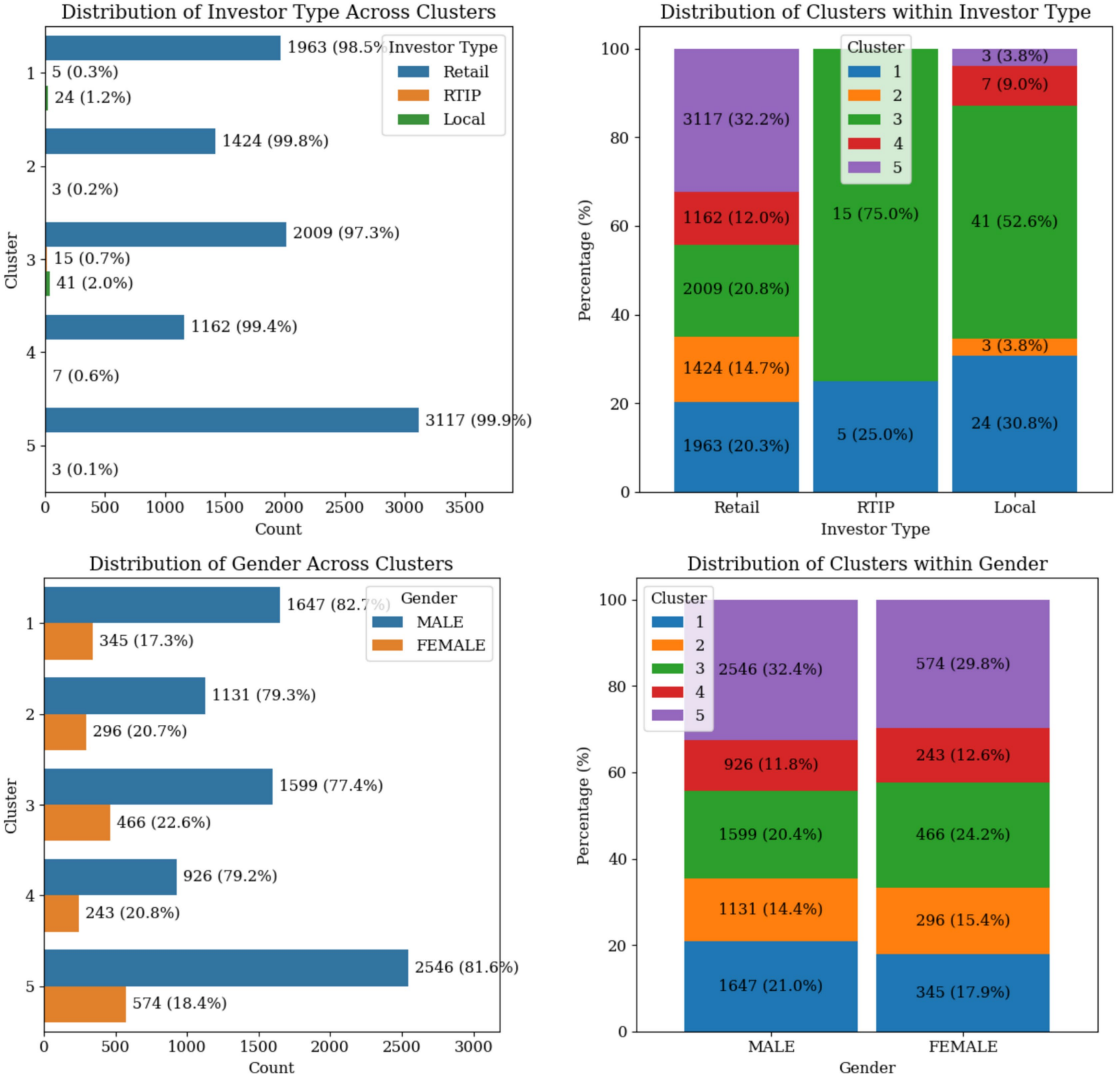
Categorical variable distributions across trader clusters showing investor type and gender.

**Figure 9:**
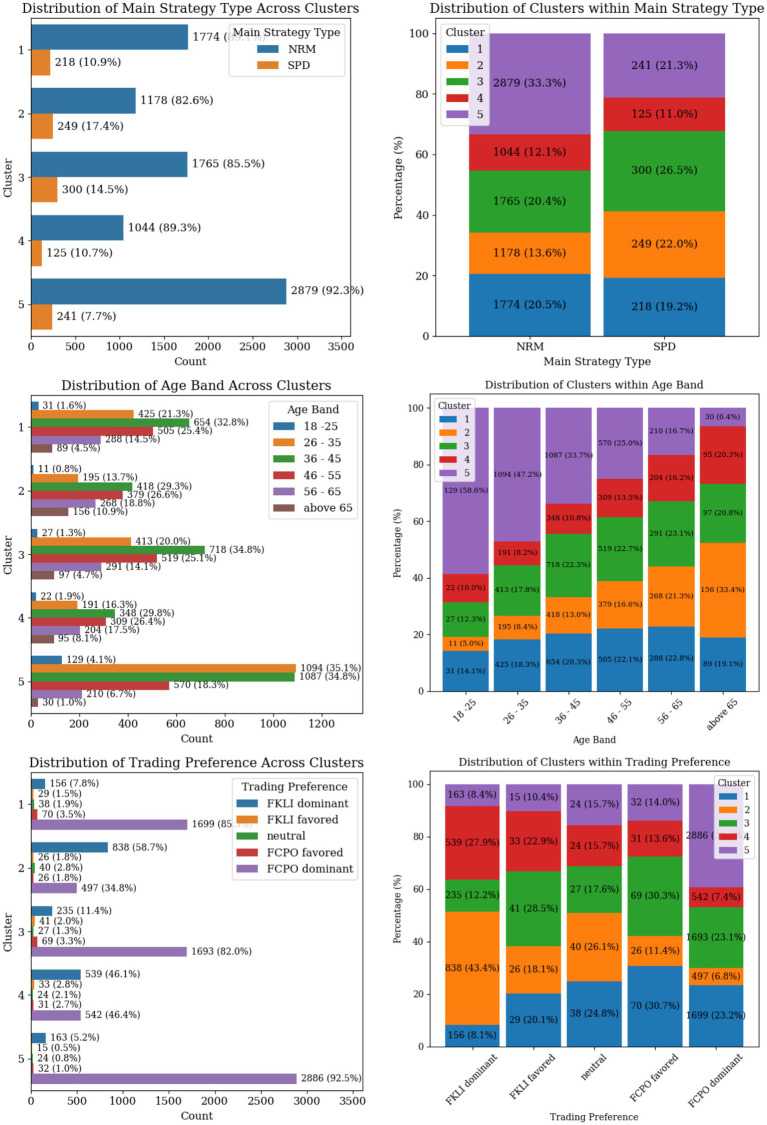
Categorical variable distributions across trader clusters showing strategy type, age band, and trading preference.

The distribution of the categorical variables across clusters and the distinct patterns in the trader profiles are found in Figures 8, 9. Retail remains the main investor type across all clusters, while RTIP and Local investors are mostly identified in Cluster 1 and 3. The proportion of males to females traders amongst all clusters is estimated roughly as 80 to 20 percent, with cluster 3 having the highest female percentage at 22%. The distribution of the Main Strategy Type exhibits a similar trend as well. Around 83–90% of the traders are mainly using the NRM strategy, with cluster 3 having a slightly higher percentage of using SPD as their main strategy.

As of the age bands, Cluster 5, comprises a significant proportion of younger traders (18–25 years and 26–35 years). This is also shown by the dominant lower Maximum Account Vintage value compared to other clusters. Cluster 2 shows an opposite age band trend compared to Cluster 5, where experienced and elder traders are concentrated there. Finally, the age groups in Cluster 1 are uniformly distributed. The trading preference within clusters shows that traders in Clusters 1, 3, and 5 are more inclined to trade FCPO when compared to Cluster 2 and 4 traders who mostly favor FKLI as their main trading preference.

A decision tree classifier is employed to determine the splitting mechanism that differentiates each cluster from the others. The pruned decision trees reveal the primary criteria for identifying and characterizing each cluster based on their trading behaviors and features (Appendix A). The characteristics of each cluster are detailed as follows.

Cluster 1 (representing 20.4% of the dataset) is defined by high trading activity, substantial traded amounts, and short holding periods. However, traders in Cluster 1 achieved consistently negative profits, indicating a high-risk and high-turnover trading strategy. The primary conditions distinguishing this cluster are a Total Realized Profit of less than RM-51.85 k, a Total Traded Amount above RM17.57 million, and a Median Number of Holding Days under 2.22 days, emphasizing frequent trading without consistent profitability.

Cluster 2 (representing 14.6% of the dataset) represents a low-risk, cautious trader profile achieving modest returns. The cluster is characterized by an Average ROI greater than 0.139%, a Median Holding Days above 0.299 days, and a Total Number of Trades below 364.5, highlighting a conservative trading strategy with moderate engagement and consistent positive returns.

Cluster 3 (representing 21.1% of the dataset) is a high-activity, high-gain trader cluster, exhibiting substantial profits and short holding periods. Traders in this group have achieved a Total Realized Profit exceeding RM40.938 thousand, a Total Traded Amount above RM15.882 million, and a Median Holding Days under 0.993 days, suggesting quick, successful trades with significant market engagement.

Cluster 4 (representing 12% of the dataset) represents cautious traders with low returns, reflecting a risk-averse strategy. This cluster is distinguished by traders achieving an Average ROI below −0.205%, a Median Holding Days above 0.203 days, and a Total Number of Trades below 306.5, indicating a conservative approach with limited profitability.

Finally, Cluster 5 (representing 31.9% of the dataset) encompasses low-activity and low-risk traders (potentially less experienced in the market). The defining criteria include a Total Number of Trades below 156.5, a Median Holding Days under 0.068 days, a Maximum Account Vintage under 3.337 years, and a Total Realized Profit under RM40.487 thousand, signifying cautious or infrequent trading and limited market exposure.

As a conclusion, the decision tree classifier results align closely with the observed boxplot distributions of the features across clusters as visualized in Figure 6. The matching patterns provide confidence that the features selected by the classifier effectively describe the unique characteristics of each cluster, enhancing the overall reliability of the analysis. Subsequently, the splitting conditions and trading behaviors revealed by the decision tree classifiers were used to label the identified clusters, providing clear and meaningful descriptions for each group. These labels capture the essence of the trading strategies, trading volume and frequency, and overall returns exhibited by the traders within each group. The labels of each identified cluster are detailed as follows.

As a conclusion, the decision tree classifier results align closely with the observed boxplot distributions of the features across clusters as visualized in Figure 6. The matching patterns provide confidence that the features selected by the classifier effectively describe the unique characteristics of each cluster, enhancing the overall reliability of the analysis. Subsequently, the splitting conditions and trading behaviors revealed by the decision tree classifiers were used to label the identified clusters, providing clear and meaningful descriptions for each group. These labels capture the essence of the trading strategies, the trading volume and frequency, and the overall returns exhibited by the traders within each group. The labels of each identified cluster are detailed as follows.

Cluster 1, “High-Frequency, High-Risk Derivative Traders with Consistent Losses,” represents a group of high-activity, high-turnover traders who prioritize frequent, short-term trades in hopes of achieving rapid gains. However, these traders often experience consistent losses. This group embodies a risk-heavy approach, where high trading volumes fail to translate into positive financial outcomes. They are also inclined to trade in FCPO products. Regulators could implement targeted interventions such as mandatory cooling-off periods or enhanced margin requirements for this segment, while brokers might benefit from automated risk controls and educational interventions to address their systematic losses. It seems to be a unique profile not commonly identified in equity market studies.

Cluster 2, “Conservative, Steady-Growth Derivative Traders,” is characterized by conservative, low-risk traders who achieve steady, modest returns, which is similar to “Conservative Investors” identified by Wang et al. These traders demonstrate a focus on minimizing risk, as reflected by their higher Average ROI, the longer Median Holding Days with low trade amounts. This group represents disciplined traders who prefer calculated strategies, avoiding excessive risk while ensuring a consistent, positive financial performance. Their cautious engagement reflects a preference for stable growth over aggressive expansion. They are also inclined to trade FKLI products with older traders. This segment presents opportunities for brokers to develop long-term investment products and advisory services, given their disciplined approach and consistent positive performance.

Cluster 3, “High-Frequency, High-Yield Derivative Traders,” comprises highly active, short-term traders who excel in generating substantial profits through quick trades. With a higher Traded Amount and shorter Median Holding Days, these traders successfully capitalize on rapid market movements as shown by their higher Realized Profit than other clusters. This group represents dynamic, successful traders who navigate the market with agility and precision, and similar to the “Active Traders” cluster described by Thompson et al. They are also inclined to trade FCPO products, while some have higher flexibility towards FKLI. Most RTIP and Local investors belong to this cluster as well. These high-performing traders could be offered premium services, lower transaction costs, and advanced trading tools by brokers, representing the most profitable client segment.

Cluster 4, “Conservative, Low-Yield Derivative Traders,” includes cautious traders who trade conservatively but yield low returns, shares similarities with “Moderate Investors” by Wang et al. Despite their steady approach (as indicated by negative Average ROI), they fail to achieve significant profitability. Their lower Number of Trades and longer Median Holding Days further reflect their preference for controlled and limited market engagement. This cluster captures the behavior of risk-averse traders who prioritize stability over aggressive strategies but struggle to convert this approach into meaningful financial gains. They are also inclined to trade in FKLI products. The negative average ROI despite cautious approaches may indicate market access barriers or information asymmetries that warrant regulatory attention and broker-provided educational support.

Cluster 5, “Cautious, Low-Activity Novice Derivative Traders,” represents low-activity traders who exhibit cautious behaviors, likely due to limited market experience. This group engages infrequently (as shown by their lower Number of Trades and Median Holding Days) while also having relatively short account histories (Maximum Account Vintage Years under 3.337 years). Their lower Realized Profit reflects modest or limited financial outcomes as well. This cluster likely consists of newer or less-engaged traders (age 18–35) who are still exploring the market or adopting a conservative approach to trading. They are also inclined to trade FCPO products. The predominance of younger, inexperienced traders in this largest cluster suggests the need for enhanced investor protection measures and mandatory financial literacy programs before derivatives trading authorization. This cluster is also similar to the “Early Savers” category identified by Thompson et al.

## Conclusions and future direction

This study explored the trading behaviors of traders in Bursa Malaysia’s derivatives markets, with a specific focus on FCPO and FKLI products. While investor segmentation has been widely studied in stock markets, this study represents a breakthrough as one of the first to apply clustering techniques to investor behavior in the derivatives market. Through the application of K-means clustering on approximately 11 million trade records, five distinct clusters were identified, “High-Frequency, High-Risk Derivative Traders with Consistent Losses,” “Conservative, Steady-Growth Derivative Trader,” “High-Frequency, High-Yield Derivative Traders,” “Conservative, Low-Yield Derivative Traders,” and “Cautious, Low-Activity Novice Derivative Traders.” The methodological approach incorporated feature engineering and IHS transformation to address extreme data variability and outliers, thereby enhancing the robustness of the clustering algorithm. The details of the clusters were discussed deeply based on the characteristics identified using a novel decision tree approach and a thorough descriptive analysis.

Future research could incorporate questionnaire-based data to establish correlations between demographic characteristics, psychological traits, and trading behaviors to relate these attributes against the identified trader clusters for more insights. However, behavioral data integration through carefully designed questionnaires would require addressing privacy and regulatory constraints inherent in financial market research with nearly 10,000 traders. Additionally, expanding the analysis to include temporal dimensions through quarterly or semi-annual segmentation would facilitate an understanding of performance trends over time. Temporal clustering analysis could be conducted with larger datasets spanning multiple years and different market cycles to ensure sufficient trader activity across all seasons while maintaining statistical validity. Future studies could also developing methodologies to accurately calculate unrealized profits, which would provide a more comprehensive view of trader performance, particularly for long-term position holders. Cross-market validation using data from other emerging derivatives markets would also enhance the generalizability of findings beyond the Malaysian context. Lastly, the current analysis does not establish whether demographic characteristics influence trading behavior clustering or merely correlate with it. Future research should incorporate formal statistical testing and expand the demographic dataset as mentioned earlier to include variables such as education level, income, trading experience, and professional background to better understand the causal relationships between trader characteristics and behavioral patterns.

## Data Availability

The datasets presented in this article are not readily available because the data used in this study were obtained under a data-sharing agreement with BURSA Malaysia. Due to the sensitive and proprietary nature of the trading data, access is restricted and the dataset is not publicly available. Interested researchers may request access directly from BURSA Malaysia; however, approval is subject to their discretion, and data access may involve administrative procedures and associated charges. The authors do not have the authority to share the dataset. Requests to access the datasets should be directed to ST, tansieowyeek@bursamalaysia.com.
