# Supplementary figures and images for: Profiling investor behavior in the Malaysian derivatives market using K-means clustering
Source: Front Artif Intell. 2025 Sep 17;8:1640776. doi: 10.3389/frai.2025.1640776 (PMC12484065; doi:10.3389/frai.2025.1640776)

Appendix A.


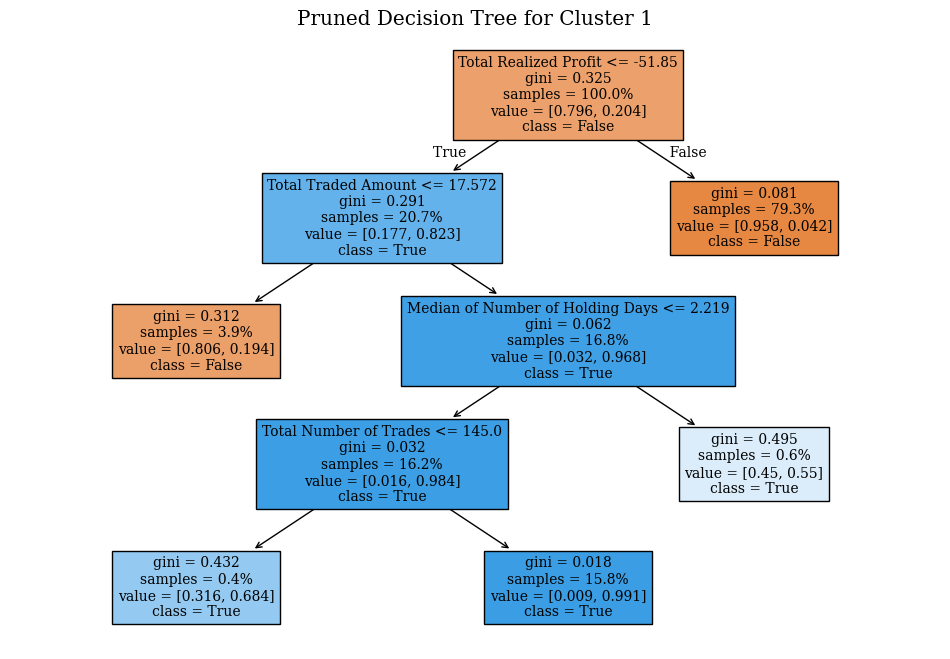


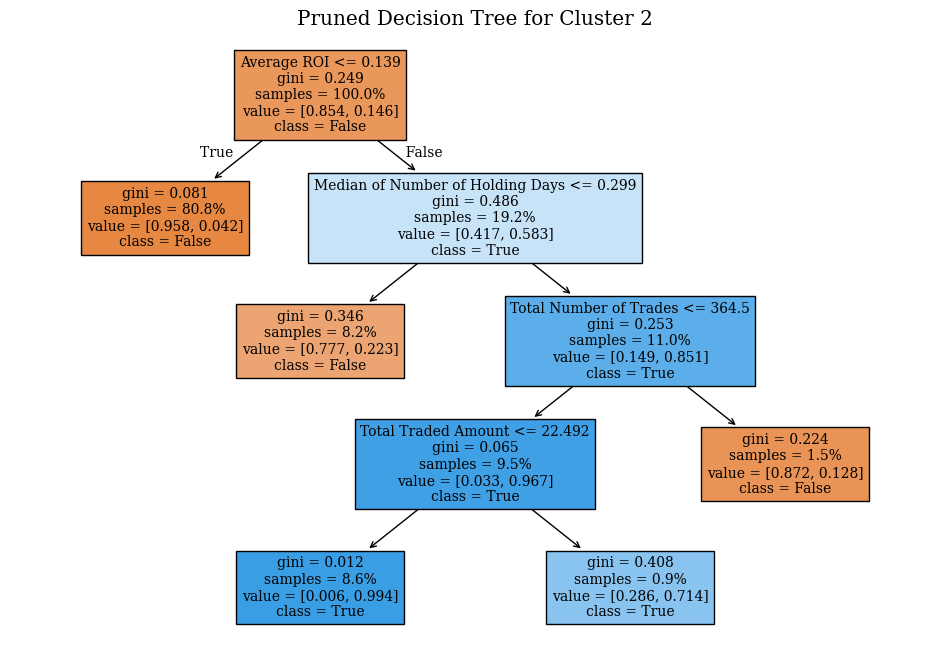


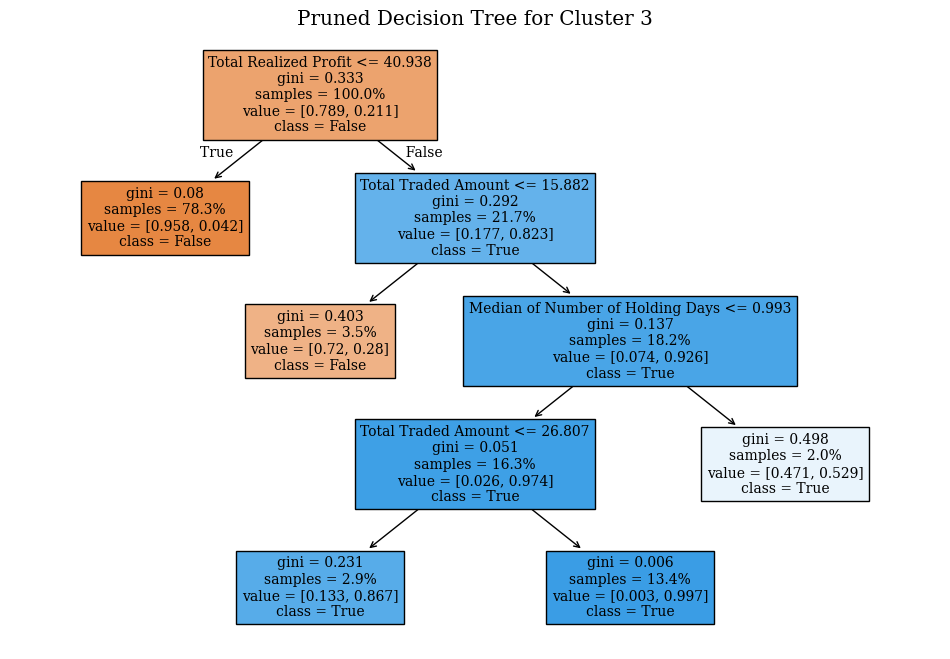

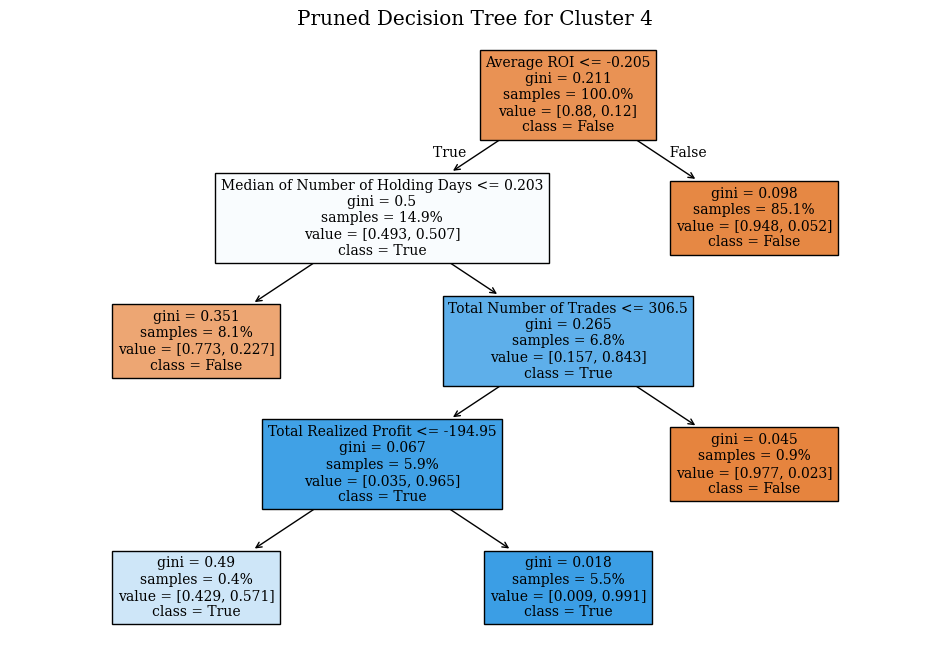


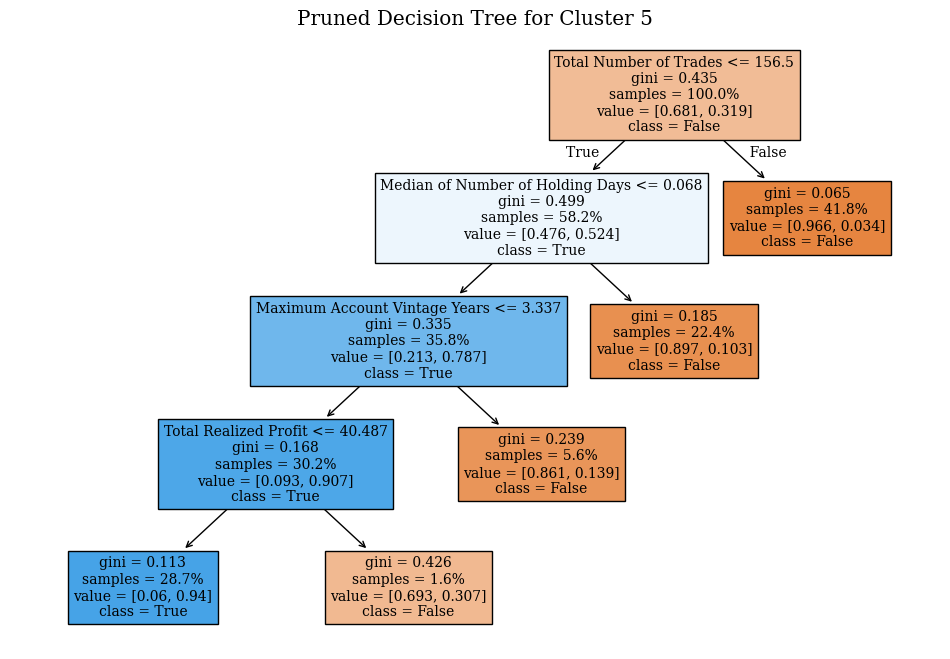

Supplement: Supplementary file 1 [file Data_Sheet_1.docx]
